# Effect of complications and reoperations on PROMIS scores for tibial plateau fractures

**DOI:** 10.1007/s00590-025-04558-0

**Published:** 2025-10-10

**Authors:** Alexa A Smitherman, Robin M Litten, Garrett N Hawkins, Doriann M Alcaide, Ryan N McIlwain, Jeffrey Clay Krout, Clay A Spitler, Joey P Johnson

**Affiliations:** 1 Heersink School of Medicine, University of Alabama at Birmingham, Birmingham, United States; 2 Department of Orthopaedic Surgery, University of Alabama at Birmingham, Birmingham, United States

**Keywords:** Tibial plateau fractures, Patient-reported outcomes, Fracture-related infection, Orthopaedic trauma, Functional outcomes

## Abstract

**Purpose:**

Tibial plateau fractures can result in significant morbidity, and complications following surgical fixation may negatively impact recovery. We aimed to evaluate whether such complications influence patient-reported outcomes at 6 months postoperatively.

**Methods:**

We conducted a retrospective cohort study at a single level I trauma center from 2022 to 2024. Adult patients who sustained a tibial plateau fracture (AO/OTA 41) treated with open reduction internal fixation (ORIF) were eligible for inclusion if they had completed Patient-Reported Outcomes Measurement Information System (PROMIS) surveys at 6 months postoperatively and had clinical follow-up confirming radiographic healing. Patients were excluded if they were under 18 years of age, lacked adequate medical record documentation, were managed non-operatively or with closed reduction percutaneous fixation, or did not complete PROMIS surveys at the 6-month time point.

The primary outcomes were PROMIS scores assessing physical function (PF), pain interference (PI), global physical health (GPH), global mental health (GMH), anxiety, and depression. Secondary outcomes included percent of normal function and Brief Resilience Scale (BRS) scores. These outcomes were compared between patients who experienced complications and those who did not.

**Results:**

A total of 106 patients were included (mean age 50.4 years; mean follow-up 261.5 days). Complications occurred in 25.5% of patients, including fracture-related infection (FRI, 10.4%), DVT/PE (7.5%), and reoperation within 6 months (11.3%). Patients with FRI had significantly lower PROMIS-PF scores at 6 months compared to those without FRI (31.5 vs. 37.4, *p* = 0.015), exceeding the MCID. Other PROMIS domains were not significantly different. Patients undergoing early reoperation prior to 6 months, or reoperation to promote bone healing at any timepoint, demonstrated lower PF scores, though these differences were not statistically significant.

**Conclusion:**

In this cohort of patients with tibial plateau fractures, FRI was associated with significantly worse physical function at 6-months as measured by the PROMIS-PF score. This difference was also clinically significant, exceeding the MCID.

**Supplementary Information:**

The online version contains supplementary material available at 10.1007/s00590-025-04558-0.

## Introduction

Tibial plateau fractures, which comprise approximately 1% of all fractures, are typically the result of high-energy trauma and represent complex injuries with significant implications on joint stability and long-term function [1]. They are often associated with damage to surrounding neurovascular and soft tissue structures, making them a potentially complex, debilitating injury [2]. In addition, they pose significant challenges for the orthopaedic surgeon, particularly in restoring the articular surface and achieving anatomic joint alignment [2–4].

Complications following these injuries including arthrofibrosis, hardware failure, nonunion, fracture-related infection (FRI), knee instability, and late osteoarthritis are common [5, 6]. Many of these complications necessitate reoperation, which may adversely affect the patient's overall wellbeing [7, 8]. There is limited literature evaluating the impact of complications following tibial plateau fractures on patient-reported outcomes (PROs). This gap is partly due to challenges in patient follow-up as well as the high variability of PROs observed among individuals with the same diagnosis [9, 10].

Patient Reported Outcome Measurement Information System (PROMIS) is a standardized system used to evaluate and compare outcomes including pain, physical function and mental health [9, 11]. The development of the PROMIS system has enabled standardized PROs that are easily comparable across patient populations, while also reducing the time and effort required from both patients and healthcare providers [9, 11]. PROMIS has become increasingly popular in orthopaedics, as it offers a standardized way to assess the perceived success of interventions and treatment protocols [12]. Although PROMIS is increasingly used in orthopaedics, most research has focused on elective procedures within subspecialties like foot and ankle, spine, and upper extremity, with limited application in the orthopaedic trauma setting [9, 12–14].

To better interpret PROMIS scores and determine whether observed changes are meaningful to patients, the concept of the minimal clinically important difference (MCID) was established [15, 16]. MCID reflects the smallest change in a PRO score that patients perceive as beneficial, offering a clinically relevant threshold for evaluating outcomes beyond statistical significance [17–19]. Incorporating MCID helps contextualize the impact of surgical complications on a patient's functional recovery and quality of life [17–19].

The purpose of this study is to evaluate if complications following fixation of tibial plateau fractures affect PROMIS scores at 6-months postoperatively. We hypothesize that patients with complications following tibial plateau open reduction internal fixation (ORIF) would demonstrate lower 6-month PROMIS scores than patients without complications, and that these differences may exceed the MCID.

## Methods

### Study design, setting, and participants

Following institutional review board approval, a retrospective review was performed to identify patients with tibial plateau fractures treated at a Level I trauma center between July 2022 and May 2024. Patients were identified from the institutional trauma database using Current Procedural Terminology (CPT) codes 27,535 and 27,536.

Inclusion criteria were patients aged 18 or older diagnosed with tibial plateau fractures (AO/OTA 41) who underwent ORIF, had clinical follow-up confirming radiographic healing, and had PROMIS scores available at the 6-month time point. Patients were excluded if they had insufficient electronic medical record (EMR) documentation, were treated non-operatively or with closed reduction percutaneous fixation (CRPF) or lacked PROMIS scores at the 6-month timepoint.

We selected 6-months as the primary time point for analysis because it represented the furthest time point from index surgery at which the majority of patients had completed outcome surveys. While survey data were available at earlier intervals (e.g., 2 weeks, 6 weeks, and 3 months), the 6-month time point provided the most complete dataset across all domains of interest, allowing for a more robust comparison between groups. Patient-reported outcomes beyond 6 months were limited due to a high rate of attrition commonly observed in trauma populations, with many patients lost to follow-up or failing to complete surveys at later time points [20–23]. As a result, 6-month scores were used as the primary indicator of mid-term functional status and patient-reported recovery.

PROMIS scores were collected using an institutional automated electronic survey sent directly to patients. The primary outcome was 6-month PROMIS scores for physical function (PF), pain interference (PI), global physical health (GPH), global mental health (GMH), anxiety and depression. Secondary outcomes included percent of normal function (0–100%) (percent of normal) and scores on the Brief Resilience Scale (BRS). The complications assessed in this study were deep vein thrombosis or pulmonary embolism (DVT/PE), unplanned reoperation within 6 months, FRI, and reoperation to promote bone healing at any time point. Patients with FRI were identified according to the consensus definition published by Metsemakers et al. in 2018 [24]. All scores (PROMIS scores, percent of normal, BRS) were compared between individuals who experienced complications and those who did not. See Supplementary Information for examples of the PROMIS questionnaire, BRS, and percent of normal surveys.

Patient demographics including age, sex, race, body mass index (BMI), tobacco use, and alcohol use were recorded. Comorbidities such as diabetes mellitus (DM) and hypertension (HTN), as well as American Society of Anesthesiologists (ASA) scores, were collected. Injury characteristics included laterality, mechanism of injury, open versus closed injury, Schatzker classification, polytrauma status, use of external fixation, and presence of ipsilateral lower extremity injuries. Clinical outcomes were also recorded. All data were obtained through a retrospective review of the electronic medical record (EMR).

### Statistical analysis

Descriptive statistics were generated to characterize the study population. Categorical variables (sex, race, tobacco use, alcohol use, comorbidities, injury characteristics) were presented as counts and percentages. Continuous variables (e.g., age, BMI, PROMIS domain scores, percent of normal, BRS) were reported as means with standard deviations. PROMIS scores, percent of normal, and BRS scores were compared using independent t-tests.

For all PROMIS domains, mean scores of each cohort (complications versus no complications) were calculated and plotted at multiple postoperative time points: 2 weeks, 6 weeks, 3 months, and 6 months. These longitudinal averages were compared between patients who experienced complications and those who did not. Statistical comparisons at each time point were performed, and corresponding *p* -values were reported to assess differences between groups over time.

All statistical analyses were performed using IBM SPSS Statistics (version 29.0.2.0). The threshold for statistical significance was defined as *p* < 0.05.

## Results

A total of 106 patients with tibial plateau fractures were included in the analysis. The average duration of follow-up was 261.5 days (range: 180–755 days). The mean age at the time of injury was 50.4 years (range: 19–81 years), and this cohort was 51.9% male. The majority of patients identified as white (70.8%). The mean BMI was 31.4 kg/m^2^ (range: 17.2–54.9 kg/m^2^), and common comorbidities included hypertension (45.3%), diabetes (19.8%), tobacco use (29.2%), and alcohol use (53.8%). Most patients were classified as ASA 2 (29.2%) or ASA 3 (63.2%) (Table 1).

**Table 1:** Demographic and clinical characteristics of 106 patients with tibial plateau fractures

Variable	
Follow-up (days), mean (SD)	261.5 (136.0)
Age, mean (SD)	50.4 (14.3)
*Sex, n (%)*	
Female	51 (48.1)
Male	55 (51.9)
*Race, n (%)*	
White	75 (70.8)
Black	26 (24.5)
Other	5 (4.7)
Body mass index (kg/m^2^), mean (SD)	31.4 (10.4)
Diabetes, n (%)	21 (19.8)
Hypertension, n (%)	48 (45.3)
Tobacco use, n (%)	31 (29.2)
Alcohol use, n (%)	57 (53.8)
*ASA score, n (%)*	
1	3 (2.8)
2	31 (29.2)
3	67 (63.2)
4	5 (4.7)

*SD* = standard deviation, *kg/m2* = kilograms per meter squared, *ASA* = American Society of Anesthesiologists

Injury characteristics and fracture patterns are summarized in Table 2. Open fractures occurred in 27 patients (25.5%), and over half (52.8%) required temporary external fixation. Forty-eight patients (45.3%) sustained polytrauma, and two-thirds (66.0%) had an ipsilateral lower extremity injury. The most common mechanisms of injury were motor vehicle collisions (39.6%), followed by falls from height (13.2%). The majority of fractures were classified as Schatzker type VI (63.2%), followed by type II (22.6%) (Table 2).

**Table 2:** Injury characteristics and fracture classification among 106 patients with tibial plateau fractures

Variable	*n* (%)
*Laterality*	
Right	49 (46.2)
Left	57 (53.8)
Open injury	27 (25.5)
External fixation	56 (52.8)
Polytrauma	48 (45.3)
Ipsilateral LE injury	70 (66.0)
*Mechanism of injury*	
MVC	42 (39.6)
MCC	10 (9.4)
FFS	13 (12.3)
FFH	14 (13.2)
GSW	3 (2.8)
Peds vs. auto	7 (6.6)
Other	17 (16.0)
*Schatzker classification*	
I	5 (4.7)
II	24 (22.6)
III	5 (4.7)
IV	5 (4.7)
V	0 (0)
VI	67 (63.2)

*MVC* = motor vehicle collision, *MCC* = motorcycle collision, *FFS* = fall from standing, *FFH* =  fall from height, *GSW* = gunshot wound, *Peds vs. auto* = pedestrian versus automobile

In our cohort of 106 patients, 27 (25.5%) experienced at least one complication following tibial plateau ORIF. Reoperations within 6 months of the index fixation occurred in 11.3% of patients, with indications including FRI, hardware removal, and knee stiffness. The incidence of FRI in this cohort was 10.4%. Reoperations to promote bone healing were performed in 6.6% of patients at any point during follow-up period. Additionally, thromboembolic events, such as DVT and PE, were observed in 7.5% of the cohort. On average, complications developed 103 days after the index surgery, ranging from 2 to 221 days (Table 3).

**Table 3:** Postoperative complications and timing observed in 106 patients with tibial plateau fractures

Variable	
Fracture-related infection, n (%)	11 (10.4)
Reoperation before 6 months, n (%)	12 (11.3)
Reoperation to promote bone healing at any time, n (%)	7 (6.6)
Deep vein thrombosis/pulmonary embolism, n (%)	8 (7.5)
Time to complication (days), mean (SD)	103.2 (69.3)

*SD* standard deviation

Patients who did not undergo reoperation reported higher PROMIS-PF scores and lower PI scores compared to those who had an unplanned reoperation within 6 months of the index surgery; however, these differences were not statistically significant (PF: 37.1 vs. 34.5, *p* = 0.210; PI: 60.8 vs. 62.1, *p* = 0.414) (Table 4). Additionally, there were no significant differences between the two groups in other PROMIS domains, including GPH, GMH, depression, anxiety, as well as percent of normal or BRS scores (Table 4).

**Table 4:** Six-month patient-reported outcomes and resilience scores by unplanned reoperation status before 6-month timepoint

Variable	Reoperation, mean (SD)	No reoperation, mean (SD)	*p*-value
PF	34.5 (7.2)	37.1 (7.0)	0.210
PI	62.1 (4.5)	60.8 (7.5)	0.414
GPH	41.9 (7.7)	40.5 (8.3)	0.587
GMH	44.6 (7.2)	45.0 (8.6)	0.873
Depression	54.0 (13.8)	54.5 (10.8)	0.879
Anxiety	55.0 (14.0)	55.6 (10.5)	0.880
Percent of normal	47.1 (17.6)	53.2 (25.1)	0.456
BRS	2.90 (1.2)	3.55 (0.7)	0.133

*SD* = standard deviation, *PF* = physical function, *PI* = pain interference, *GPH* = global physical health, *GMH* = global mental health, *BRS* = brief resilience scale

Patients with FRI demonstrated statistically significant lower 6-month PROMIS-PF scores compared to those without FRI (31.5 vs. 37.4, *p* = 0.015; Table 5). Thorne et al. reported the MCID for PROMIS-PF scores in patients with tibial plateau fractures to range from 3.93 to 4.85 [15]. In our cohort, the mean difference in PROMIS-PF scores for patients with FRI was 5.9, exceeding this established MCID threshold. No statistically significant differences were observed in other PROMIS domains, percent of normal, or BRS scores between these two groups (Table 5). Similarly, patients who experienced thromboembolic complications did not demonstrate any statistically significant differences in PROMIS scores, percent of normal, or BRS scores (Table 6).

**Table 5:** Six-month patient-reported outcomes and resilience scores by FRI status

Variable	FRI, mean (SD)	No FRI, mean (SD)	*p*-value
PF	31.5 (8.1)	37.4 (6.7)	0.015
PI	60.8 3.3)	61.0 (7.5)	0.941
GPH	42.6 (7.9)	40.5 (8.3)	0.447
GMH	45.4 (6.1)	45.0 (8.6)	0.847
Depression	52.9 (12.4)	54.6 (11.0)	0.663
Anxiety	55.3 (13.0)	55.5 (10.8)	0.961
Percent of normal	57.6 (24.7)	52.1 (24.5)	0.545
BRS	3.6 (1.2)	3.5 (0.7)	0.933

*FRI* = fracture-related infection, *SD* = standard deviation, *PF* = physical function, *PI* = pain interference, *GPH* = global physical health, *GMH* = global mental health, *BRS* = brief resilience scale

**Table 6:** Six-month patient-reported outcomes and resilience scores stratified by presence of thromboembolic complications

Variable	DVT/PE, mean (SD)	No DVT/PE, mean (SD)	*p*-value
PF	37.7 (7.2)	36.8 (7.1)	0.364
PI	59.8 (8.6)	61.0 (7.2)	0.319
GPH	42.6 (9.6)	40.5 (8.1)	0.247
GMH	48.9 (7.3)	44.6 (8.4)	0.163
Depression	53.1 (10.6)	54.6 (11.2)	0.720
Anxiety	59.3 (9.5)	55.2 (11.0)	0.306
Percent of normal	63.3 (28.4)	51.8 (24.1)	0.230
BRS	3.5 (0.7)	3.5 (0.8)	0.908

*DVT/PE* = deep vein thrombosis/pulmonary embolism, *SD* = standard deviation, *PF* = physical function, *PI* = pain interference, *GPH* = global physical health, *GMH* = global mental health, *BRS* = brief resilience scale

Seven patients (6.6%) underwent reoperation to promote bone healing throughout the entire follow-up period. When comparing their 6-month PROMIS scores to those who did not have a reoperation to promote bone healing, patients who required this procedure demonstrated lower PROMIS-PF scores compared to those who did not (33.3 vs. 37.1, respectively). However, the difference in PF scores, as well as other PROMIS domains, did not reach statistical significance. Percent of normal and BRS scores were also similar between the groups (Table 7).

**Table 7:** Six-month patient-reported outcomes and resilience scores stratified by reoperation to promote bone healing (RPBH)

Variable	RPBH, mean (SD)	No RPBH, mean (SD)	*p*-value
PF	33.3 (10.4)	37.1 (6.7)	0.166
PI	60.4 (10.9)	61.0 (7.0)	0.836
GPH	40.3 (8.8)	40.7 (8.2)	0.890
GMH	40.6 (10.1)	45.2 (8.2)	0.158
Depression	58.1 (12.0)	54.2 (11.1)	0.373
Anxiety	58.3 (12.2)	55.3 (10.9)	0.484
Percent of normal	54.8 (33.3)	52.4 (24.0)	0.817
BRS	3.17 (0.7)	3.51 (0.8)	0.299

*SD*  =standard deviation, *PF * =physical function, *PI* = pain interference, *GPH*  =global physical health, *GMH* =global mental health, *BRS*  = brief resilience scale

Because all seven patients who underwent reoperation to promote bone healing did so after the 6-month postoperative time point, we compared the mean 6-month PROMIS scores, percent of normal, and BRS scores of this cohort to mean scores documented at the 1-year follow-up (Table 8). Overall, there were no statistically significant differences observed between 6-month and 1-year scores across any of the measured domains. PROMIS-PI demonstrated an increase, though it was not statistically significant (60.4–64.5; *p* = 0.538). Notably, the percent of normal reported by patients decreased from 54.8% at 6 months to 40.8% at 1 year, although this change was not statistically significant (*p* = 0.481). Resilience as measured by the BRS increased slightly, though this was also not significant (3.17 vs. 3.3; *p* = 0.664) (Table 8).

**Table 8:** Comparison of six-month and 1-year patient-reported outcomes and resilience scores of patients who underwent reoperation to promote bone healing (RPBH)

Variable	6-month score, mean (SD)	1-year score, mean (SD)	*p*-value
PF	33.3 (10.4)	34.7 (2.9)	0.798
PI	60.4 (10.9)	64.5 (8.2)	0.538
GPH	40.3 (8.8)	38.4 (5.9)	0.693
GMH	40.6 (10.1)	43.1 (4.6)	0.623
Depression	58.1 (12.0)	56.8 (6.6)	0.846
Anxiety	58.3 (12.2)	59.6 (5.70	0.855
Percent of normal	54.8 (33.3)	40.8 (22.0)	0.481
BRS	3.17 (0.7)	3.3 (0.3)	0.664

*SD* = standard deviation, *PF* = physical function, *PI* = pain interference, *GPH* = global physical health, *GMH* = global mental health, *BRS* = brief resilience scale

**Fig. 1:**
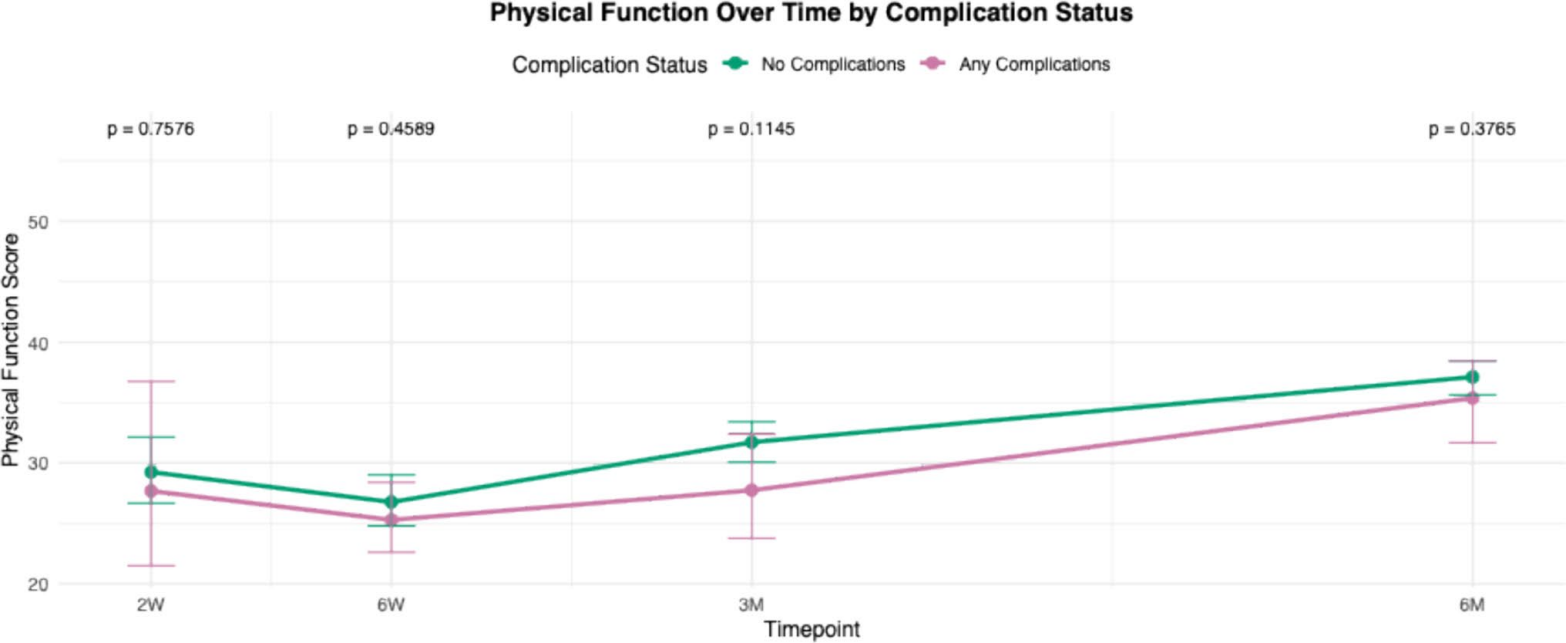
Longitudinal comparison of PROMIS-PF scores in patients with and without complications throughout 6 months following index surgery. PROMIS = Patient-Reported Outcomes Measurement Information System, PF = physical function

**Fig. 2:**
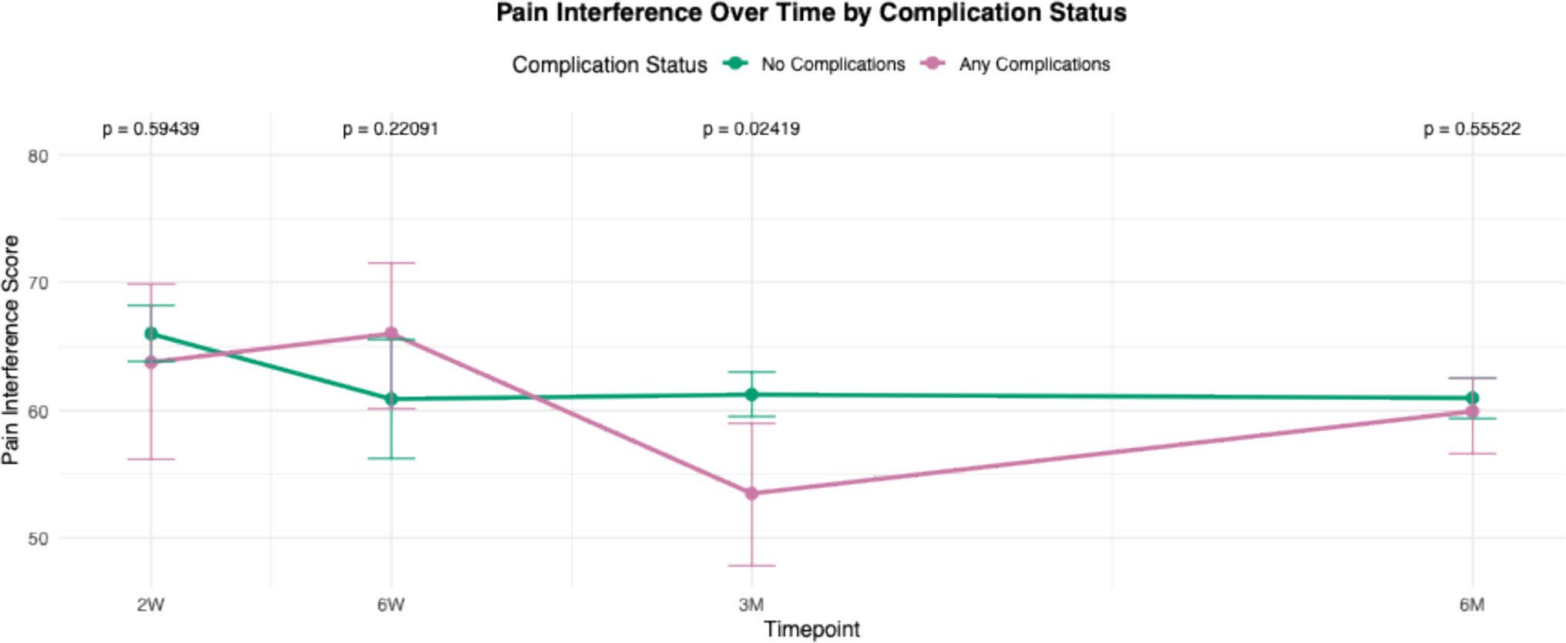
Longitudinal comparison of PROMIS-PI scores in patients with and without complications throughout 6 months following index surgery. PROMIS = Patient-Reported Outcomes Measurement Information System, PI = pain interference

**Fig. 3:**
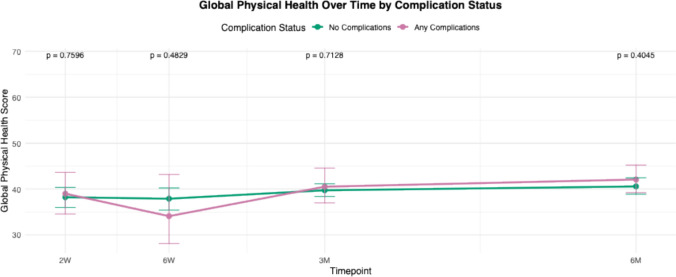
Longitudinal comparison of PROMIS-GPH scores in patients with and without complications throughout 6 months following index surgery. PROMIS = Patient-Reported Outcomes Measurement Information System, GPH = global physical health

**Fig. 4:**
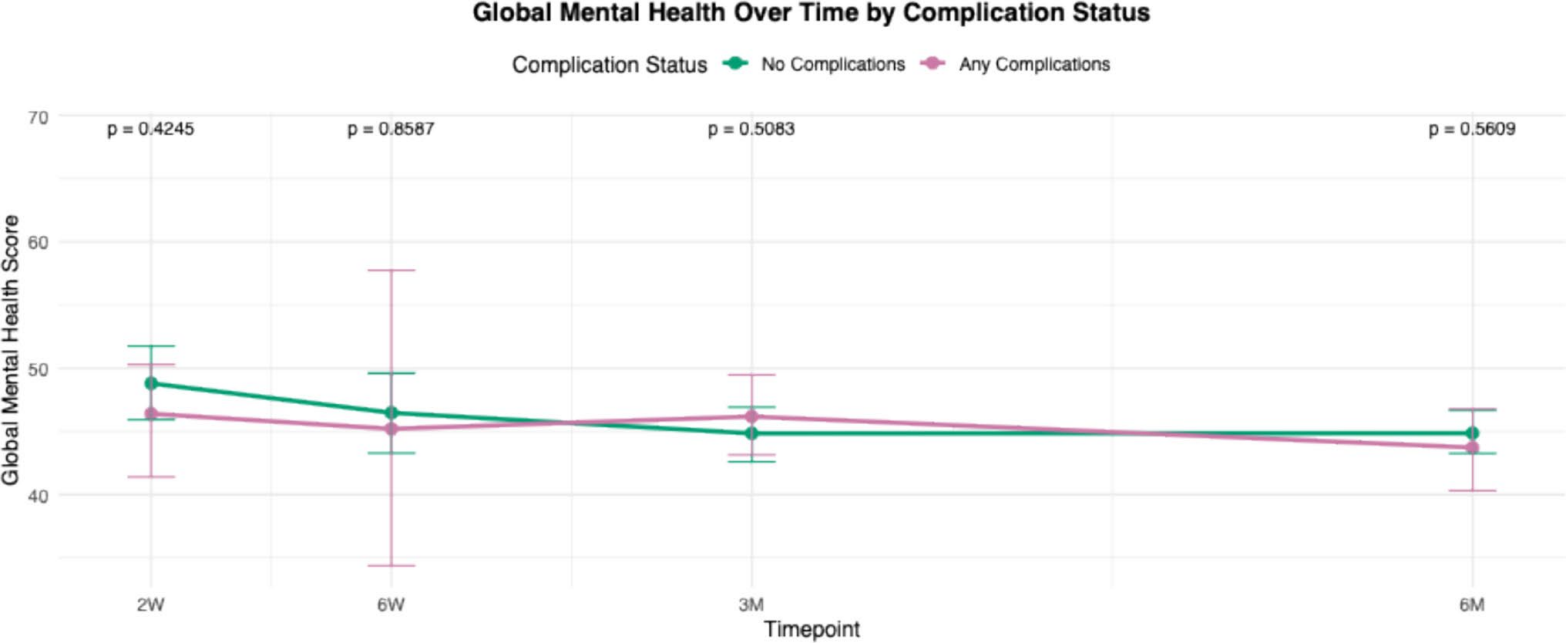
Longitudinal comparison of PROMIS-GMH scores in patients with and without complications throughout 6 months following index surgery. PROMIS = Patient-Reported Outcomes Measurement Information System, GMH = global mental health

**Fig. 5:**
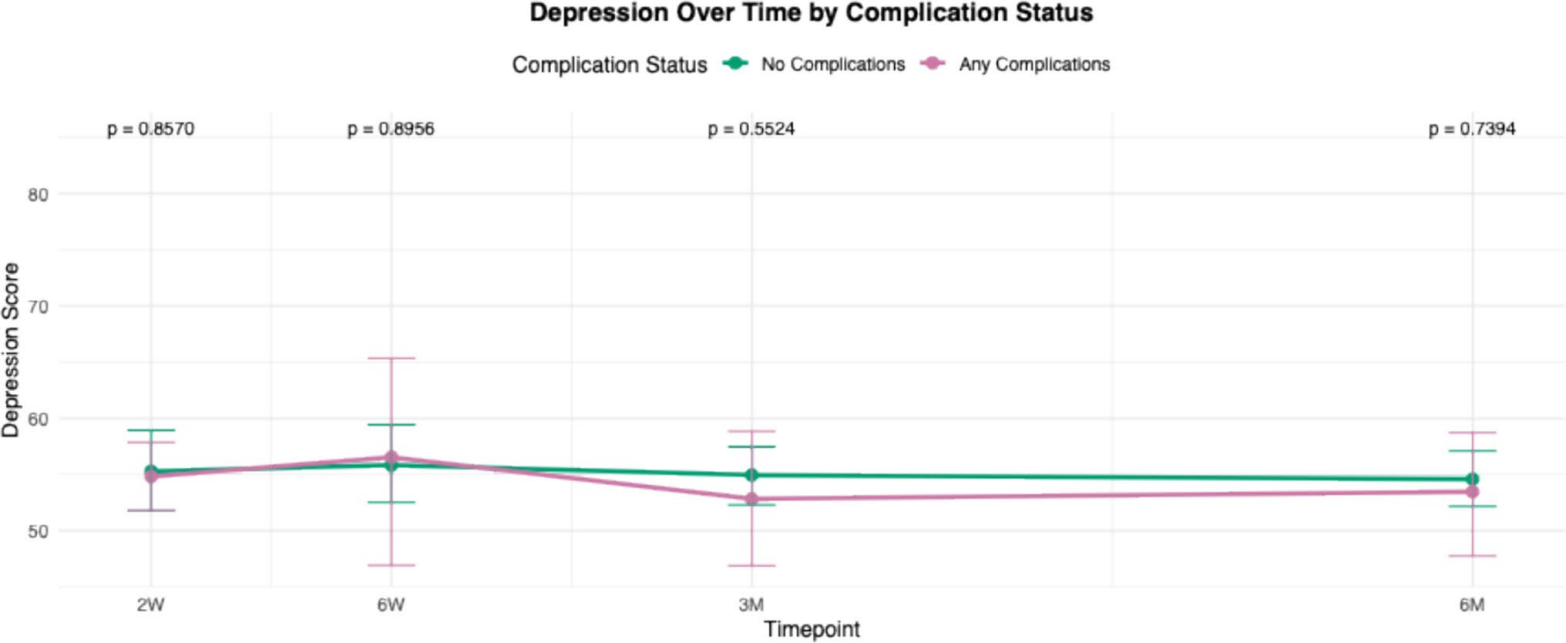
Longitudinal comparison of PROMIS-Depression scores in patients with and without complications throughout 6 months following index surgery. PROMIS = Patient-Reported Outcomes Measurement Information System

**Fig. 6:**
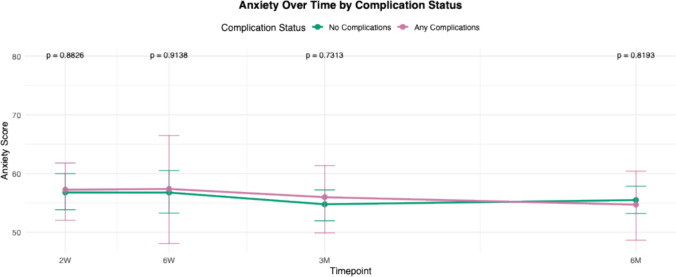
Longitudinal comparison of PROMIS-Anxiety scores in patients with and without complications throughout 6 months following index surgery. PROMIS = Patient-Reported Outcomes Measurement Information System

Trend lines for PROMIS scores in patients with any complications were compared to those without complications from 2 weeks to 6 months (Figs. 1, 2, 3, 4, 5and 6). These showed similar trends and scores in PROMIS-PF, GPH, GMH, anxiety or depression at all timepoints. A significant difference in PROMIS-PI trends was observed, with patients who experienced complications reporting lower PI scores at the 3-month timepoint (*p* = 0.024). However, this difference was no longer evident at 6 months (*p* = 0.555) (Fig. 2).

## Discussion

FRI was associated with significantly lower PROMIS-PF scores in patients with tibial plateau fractures at 6 months. Furthermore, while not statistically significant, patients who underwent reoperation to promote bone healing at any point, as well as those who required reoperation within 6 months, demonstrated a trend toward lower PROMIS-PF scores. No trends in other PROMIS domains, percent of normal, or BRS outcomes were observed across the different complication subtypes.

The 6-month reoperation rate of 11.3% following the index surgery aligns with previously reported rates for tibial plateau fractures, which range from 10 to 30% [25–28]. This study did not find any statistically significant differences in PROs amongst patients that required reoperation and those that did not at the 6-month time point. Although PROMIS is a relatively new patient-reported outcome measure, prior research has shown that patients with tibial plateau fractures who undergo reoperation experience worse functional outcomes at 6 months, as measured by the Short Musculoskeletal Function Assessment (SMFA). [8]. While not a direct comparison, studies have shown a correlation between PROMIS-PF and SMFA scores in patients with lower extremity fractures, suggesting these measures reflect similar functional outcomes [29].

The FRI rate in this study was 10.4%, which is lower than rates previously reported in the literature (12.0–17.0%) [26–28, 30]. In this cohort, patients with FRI demonstrated lower physical function scores compared to those without infection. This finding is consistent with a previous study by O’Neill et al., which reported that infection was associated with worse PROMIS-PF scores [31].

Thorne et al. established the MCID in PROMIS-PF scores of patients with tibial plateaus to be from 3.93 to 4.85 [15]. In our study, the average score difference for patients with FRI exceeded this MCID (5.9). The MCID was not met for patients with reoperations before 6 months (2.6) or reoperations to promote bone healing at any time point (3.8). These findings, in conjunction with prior work by O’Neill, suggest that infection has a clinically significant negative impact on physical function following tibial plateau fractures [15, 31].

The 6.6% rate of reoperation to promote bone healing (RPBH) at any time point during the follow up period aligns with previously reported rates in the literature for tibial plateaus (6.0–9.0%) [26–28]. This study also found no difference in PROMIS scores for patients with and without RPBH. Although differences in PROMIS-PF and PROMIS-PI scores have been previously reported in patients undergoing RPBH after tibial shaft fractures, there is limited data on PROs or PROMIS scores specifically in tibial plateau fractures [32]. Furthermore, a systematic review of reoperations to promote bone healing following tibial plateau fractures has emphasized the variability in PRO measures used across studies [33].

In addition to physical function, this study also assessed mental health outcomes in patients with tibial plateau fractures who experienced various complications. Although mental health domains such as global mental health, depression, and anxiety are not commonly reported in orthopedic research, they are important in the orthopedic trauma population, as prior studies have demonstrated that post-trauma depression and anxiety can diminish quality of life, hinder physical recovery, extend pain medication use, and increase hospital readmission rates [12, 34–36]. O’Neill et al. similarly reported worse mental health scores in patients with tibial plateau infections [31]. While this study did not find significant differences in mental health outcomes, it remains important to recognize the potential impact of complications on patients’ mental wellbeing, recovery, and overall health after tibial plateau fractures.

### Limitations

This study has limitations that should be considered when interpreting these findings, including those inherent to its retrospective design. This study was conducted at a single institution, which may limit generalizability to other settings with different patient populations, clinical protocols, or tibial plateau management practices. Additionally, the analysis focused on selected complications and only included reoperations occurring within 6 months post-index surgery due to PROMIS score availability, which may underestimate the impact of later complications on patient-reported outcomes. Finally, variability in PROMIS survey completion and timing may have introduced response bias and limited the ability to capture all fluctuations in recovery over time.

## Conclusion

In this cohort of patients with tibial plateau fractures, FRI was associated with significantly worse physical function at 6-months as measured by the PROMIS-PF score. This difference was also clinically significant, exceeding the MCID. Furthermore, while not statistically significant, patients who underwent reoperation to promote bone healing at any point, as well as those who required reoperation within 6 months, demonstrated a trend toward lower 6-month PROMIS-PF scores. No other PROMIS domains, as well as BRS and percent of normal, differed significantly across complication subtypes.

## Supplementary Information

Below is the link to the electronic supplementary material.

Supplementary file1 (PDF 1266 kb)

## Data Availability

No datasets were generated or analysed during the current study.
