# Supplementary material for: Effect of complications and reoperations on PROMIS scores for tibial plateau fractures
Source: Eur J Orthop Surg Traumatol. 2025 Oct 10;35(1):430. doi: 10.1007/s00590-025-04558-0 (PMC12513888; doi:10.1007/s00590-025-04558-0)

UAB MEDICINE | Orthopaedic Surgery

Patient-Reported Outcomes Measurement Information System (PROMIS) Surveys

PROMIS Global Health

Please respond to each question or statement by marking one box per row.

Scale for Global01 to Global06 and Global05:
5 = Excellent
4 = Very good
3 = Good
2 = Fair
1 = Poor

Global01
In general, would you say your health is:
5 □
4 □
3 □
2 □
1 □

Global02
In general, would you say your quality of life is:
5 □
4 □
3 □
2 □
1 □

Global03
In general, how would you rate your physical health?
5 □
4 □
3 □
2 □
1 □

Global04
In general, how would you rate your mental health, including your mood and your ability to think?
5 □
4 □
3 □

2 □
1 □

Global05
In general, how would you rate your satisfaction with your social activities and relationships?
5 □
4 □
3 □
2 □
1 □

Global09r
In general, please rate how well you carry out your usual social activities and roles.
(This includes activities at home, at work and in your community, and responsibilities as a parent, child, spouse, employee, friend, etc.)
5 □ Completely
4 □ Mostly
3 □ Moderately
2 □ A little
1 □ Not at all

Global06
To what extent are you able to carry out your everyday physical activities such as walking, climbing stairs, carrying groceries, or moving a chair?
5 □
4 □
3 □
2 □
1 □

In the past 7 days…
How often have you been bothered by emotional problems such as feeling anxious, depressed or irritable?
Never □
Rarely □
Sometimes □
Often □
Always □
(Scale 5 to 1)

Global08r

How would you rate your fatigue on average?

5 □

4 □

3 □

2 □

1 □

Global07r

How would you rate your pain on average?

0 □ No pain

1 □

2 □

3 □

4 □

5 □

6 □

7 □

8 □

9 □

10 □ Worst pain imaginable

PROMIS Emotional Distress - Anxiety

Please respond to each item by marking one box per row.

In the past 7 days…

Scale:

1 = Never 2 = Rarely 3 = Sometimes 4 = Often 5 = Always

EDANX01 I felt fearful

□ 1 □ 2 □ 3 □ 4 □ 5

EDANX02 I felt frightened

□ 1 □ 2 □ 3 □ 4 □ 5

EDANX03 It scared me when I felt nervous

□ 1 □ 2 □ 3 □ 4 □ 5

EDANX05 I felt anxious

□ 1 □ 2 □ 3 □ 4 □ 5

EDANX07 I felt like I needed help for my anxiety

□ 1 □ 2 □ 3 □ 4 □ 5

EDANX08 I was concerned about my mental health

□ 1 □ 2 □ 3 □ 4 □ 5

EDANX12 I felt upset

□ 1 □ 2 □ 3 □ 4 □ 5

EDANX13 I had a racing or pounding heart

□ 1 □ 2 □ 3 □ 4 □ 5

EDANX16 I was anxious if my normal routine was disturbed

□ 1 □ 2 □ 3 □ 4 □ 5

EDANX18 I had sudden feelings of panic

□ 1 □ 2 □ 3 □ 4 □ 5

EDANX20 I was easily startled

□ 1 □ 2 □ 3 □ 4 □ 5

EDANX21 I had trouble paying attention

□ 1 □ 2 □ 3 □ 4 □ 5

EDANX24 I avoided public places or activities

□ 1 □ 2 □ 3 □ 4 □ 5

EDANX26 I felt fidgety
□ 1 □ 2 □ 3 □ 4 □ 5

EDANX27 I felt something awful would happen
□ 1 □ 2 □ 3 □ 4 □ 5

EDANX30 I felt worried
□ 1 □ 2 □ 3 □ 4 □ 5

EDANX33 I felt terrified
□ 1 □ 2 □ 3 □ 4 □ 5

EDANX37 I worried about other people's reactions to me
□ 1 □ 2 □ 3 □ 4 □ 5

EDANX40 I found it hard to focus on anything other than my anxiety
□ 1 □ 2 □ 3 □ 4 □ 5

EDANX41 My worries overwhelmed me
□ 1 □ 2 □ 3 □ 4 □ 5

EDANX44 I had twitching or trembling muscles
□ 1 □ 2 □ 3 □ 4 □ 5

EDANX46 I felt nervous
□ 1 □ 2 □ 3 □ 4 □ 5

EDANX47 I felt indecisive
□ 1 □ 2 □ 3 □ 4 □ 5

EDANX48 Many situations made me worry
□ 1 □ 2 □ 3 □ 4 □ 5

EDANX49 I had difficulty sleeping
□ 1 □ 2 □ 3 □ 4 □ 5

EDANX51 I had trouble relaxing
□ 1 □ 2 □ 3 □ 4 □ 5

EDANX53 I felt uneasy
□ 1 □ 2 □ 3 □ 4 □ 5

EDANX54 I felt tense
□ 1 □ 2 □ 3 □ 4 □ 5

EDANX55 I had difficulty calming down
□ 1 □ 2 □ 3 □ 4 □ 5

UAB MEDICINE | Orthopaedic Surgery

PROMIS - Emotional Distress: Depression

Please respond to each item by marking one box per row.
In the past 7 days…
Scale:
1 = Never 2 = Rarely 3 = Sometimes 4 = Often 5 = Always

EDDEP04 I felt worthless
□ 1 □ 2 □ 3 □ 4 □ 5

EDDEP05 I felt that I had nothing to look forward to
□ 1 □ 2 □ 3 □ 4 □ 5

EDDEP06 I felt helpless
□ 1 □ 2 □ 3 □ 4 □ 5

EDDEP07 I withdrew from other people
□ 1 □ 2 □ 3 □ 4 □ 5

EDDEP09 I felt that nothing could cheer me up
□ 1 □ 2 □ 3 □ 4 □ 5

EDDEP14 I felt that I was not as good as other people
□ 1 □ 2 □ 3 □ 4 □ 5

EDDEP17 I felt sad
□ 1 □ 2 □ 3 □ 4 □ 5

EDDEP19 I felt that I wanted to give up on everything
□ 1 □ 2 □ 3 □ 4 □ 5

EDDEP21 I felt that I was to blame for things
□ 1 □ 2 □ 3 □ 4 □ 5

EDDEP22 I felt like a failure
□ 1 □ 2 □ 3 □ 4 □ 5

EDDEP23 I had trouble feeling close to people
□ 1 □ 2 □ 3 □ 4 □ 5

EDDEP26 I felt disappointed in myself
□ 1 □ 2 □ 3 □ 4 □ 5

EDDEP27 I felt that I was not needed
□ 1 □ 2 □ 3 □ 4 □ 5

EDDEP28 I felt lonely
□ 1 □ 2 □ 3 □ 4 □ 5

EDDEP29 I felt depressed
□ 1 □ 2 □ 3 □ 4 □ 5

EDDEP30 I had trouble making decisions
□ 1 □ 2 □ 3 □ 4 □ 5

EDDEP31 I felt discouraged about the future
□ 1 □ 2 □ 3 □ 4 □ 5

EDDEP35 I found that things in my life were overwhelming
□ 1 □ 2 □ 3 □ 4 □ 5

EDDEP36 I felt unhappy
□ 1 □ 2 □ 3 □ 4 □ 5

EDDEP39 I felt I had no reason for living
□ 1 □ 2 □ 3 □ 4 □ 5

EDDEP41 I felt hopeless
□ 1 □ 2 □ 3 □ 4 □ 5

EDDEP42 I felt ignored by people
□ 1 □ 2 □ 3 □ 4 □ 5

EDDEP44 I felt upset for no reason
□ 1 □ 2 □ 3 □ 4 □ 5

EDDEP45 I felt that nothing was interesting
□ 1 □ 2 □ 3 □ 4 □ 5

EDDEP46 I felt pessimistic
□ 1 □ 2 □ 3 □ 4 □ 5

EDDEP48 I felt that my life was empty
□ 1 □ 2 □ 3 □ 4 □ 5

EDDEP50 I felt guilty
□ 1 □ 2 □ 3 □ 4 □ 5

EDDEP54 I felt emotionally exhausted
□ 1 □ 2 □ 3 □ 4 □ 5

UAB MEDICINE | Orthopaedic Surgery

PROMIS Pain Interference

Please respond to each item by marking one box per row.
Timeframe: In the past 7 days

Scale A (Interference Frequency & Intensity):
1 = Not at all 2 = A little bit 3 = Somewhat 4 = Quite a bit 5 = Very much

---

PAININ1 How difficult was it for you to take in new information because of pain?
□ 1 □ 2 □ 3 □ 4 □ 5

PAININ3 How much did pain interfere with your enjoyment of life?
□ 1 □ 2 □ 3 □ 4 □ 5

PAININ5 How much did pain interfere with your ability to participate in leisure activities?
□ 1 □ 2 □ 3 □ 4 □ 5

PAININ6 How much did pain interfere with your close personal relationships?
□ 1 □ 2 □ 3 □ 4 □ 5

PAININ8 How much did pain interfere with your ability to concentrate?
□ 1 □ 2 □ 3 □ 4 □ 5

PAININ9 How much did pain interfere with your day-to-day activities?
□ 1 □ 2 □ 3 □ 4 □ 5

PAININ10 How much did pain interfere with your enjoyment of recreational activities?
□ 1 □ 2 □ 3 □ 4 □ 5

PAININ11 How often did you feel emotionally tense because of your pain?
□ 1 □ 2 □ 3 □ 4 □ 5

PAININ12 How much did pain interfere with the things you usually do for fun?
□ 1 □ 2 □ 3 □ 4 □ 5

PAININ13 How much did pain interfere with your family life?
□ 1 □ 2 □ 3 □ 4 □ 5

PAININ17 How much did pain interfere with your relationships with other people?
□ 1 □ 2 □ 3 □ 4 □ 5

PAININ18 How much did pain interfere with your ability to work (including work at home)?
□ 1 □ 2 □ 3 □ 4 □ 5

PAININ19 How much did pain make it difficult to fall asleep?
□ 1 □ 2 □ 3 □ 4 □ 5

PAININ20 How much did pain feel like a burden to you?
□ 1 □ 2 □ 3 □ 4 □ 5

PAININ22 How much did pain interfere with work around the home?
□ 1 □ 2 □ 3 □ 4 □ 5

PAININ31 How much did pain interfere with your ability to participate in social activities?
□ 1 □ 2 □ 3 □ 4 □ 5

PAININ34 How much did pain interfere with your household chores?
□ 1 □ 2 □ 3 □ 4 □ 5

PAININ35 How much did pain interfere with your ability to make trips from home that kept you gone for more than 2 hours?
□ 1 □ 2 □ 3 □ 4 □ 5

PAININ36 How much did pain interfere with your enjoyment of social activities?
□ 1 □ 2 □ 3 □ 4 □ 5

PAININ48 How much did pain interfere with your ability to do household chores?
□ 1 □ 2 □ 3 □ 4 □ 5

PAININ49 How much did pain interfere with your ability to remember things?
□ 1 □ 2 □ 3 □ 4 □ 5

PAININ56 How irritable did you feel because of pain?
□ 1 □ 2 □ 3 □ 4 □ 5

PAININ14 How much did pain interfere with doing your tasks away from home (e.g., getting groceries, running errands)?
□ 1 □ 2 □ 3 □ 4 □ 5

Scale B (Frequency-based Impact):
1 = Never 2 = Rarely 3 = Sometimes 4 = Often 5 = Always

PAININ16 How often did pain make you feel depressed?
□ 1 □ 2 □ 3 □ 4 □ 5

PAININ24 How often was pain distressing to you?
□ 1 □ 2 □ 3 □ 4 □ 5

UAB MEDICINE | Orthopaedic Surgery

PAININ26 How often did pain keep you from socializing with others?
□ 1 □ 2 □ 3 □ 4 □ 5

PAININ29 How often was your pain so severe you could think of nothing else?
□ 1 □ 2 □ 3 □ 4 □ 5

PAININ32 How often did pain make you feel discouraged?
□ 1 □ 2 □ 3 □ 4 □ 5

PAININ37 How often did pain make you feel anxious?
□ 1 □ 2 □ 3 □ 4 □ 5

PAININ38 How often did you avoid social activities because it might make you hurt more?
□ 1 □ 2 □ 3 □ 4 □ 5

PAININ40 How often did pain prevent you from walking more than 1 mile?
□ 1 □ 2 □ 3 □ 4 □ 5

PAININ42 How often did pain prevent you from standing for more than one hour?
□ 1 □ 2 □ 3 □ 4 □ 5

PAININ46 How often did pain make it difficult for you to plan social activities?
□ 1 □ 2 □ 3 □ 4 □ 5

PAININ47 How often did pain prevent you from standing for more than 30 minutes?
□ 1 □ 2 □ 3 □ 4 □ 5

PAININ50 How often did pain prevent you from sitting for more than 30 minutes?
□ 1 □ 2 □ 3 □ 4 □ 5

PAININ51 How often did pain prevent you from sitting for more than 10 minutes?
□ 1 □ 2 □ 3 □ 4 □ 5

PAININ52 How often was it hard to plan social activities because you didn't know if you would be in pain?
□ 1 □ 2 □ 3 □ 4 □ 5

PAININ53 How often did pain restrict your social life to your home?
□ 1 □ 2 □ 3 □ 4 □ 5

PAININ55 How often did pain prevent you from sitting for more than one hour?
□ 1 □ 2 □ 3 □ 4 □ 5

PAININ54 How often did pain keep you from getting into a standing position?
□ 1 = Never 2 = Once a week or less 3 = Once every few days 4 = Once a day 5 = Every few hours

UAB MEDICINE | Orthopaedic Surgery

PROMIS Physical Function

Response Options:

- 5 – Without any difficulty
- 4 – With a little difficulty
- 3 – With some difficulty
- 2 – With much difficulty
- 1 – Unable to do

PROMIS Physical Function Items:

PFA8 – Are you able to move a chair from one room to another?
PFA9 – Are you able to bend down and pick up clothing from the floor?
PFA10 – Are you able to stand for one hour?
PFA11 – Are you able to do chores such as vacuuming or yard work?
PFA12 – Are you able to push open a heavy door?
PFA13 – Are you able to exercise for an hour?
PFA14r1 – Are you able to carry a heavy object (over 10 pounds / 5 kg)?
PFA15 – Are you able to stand up from an armless straight chair?
PFA16r1 – Are you able to dress yourself, including tying shoelaces and buttoning your clothes?
PFA17 – Are you able to reach into a high cupboard?
PFA18 – Are you able to use a hammer to pound a nail?
PFA19r1 – Are you able to run or jog for two miles (3 km)?
PFA20 – Are you able to cut your food using eating utensils?
PFA21 – Are you able to go up and down stairs at a normal pace?
PFA23 – Are you able to go for a walk of at least 15 minutes?
PFA27 – Are you able to run on uneven ground?
PFA28 – Are you able to open a can with a hand can opener?
PFA29r1 – Are you able to pull heavy objects (10 pounds / 5 kg) towards yourself?
PFA30 – Are you able to step up and down curbs?
PFA31r1 – Are you able to get up from the floor from lying on your back without help?
PFA32 – Are you able to stand with your knees straight?
PFA33 – Are you able to exercise hard for half an hour?
PFA34 – Are you able to wash your back?
PFA35 – Are you able to open and close a zipper?
PFA36 – Are you able to put on and take off a coat or jacket?
PFA37 – Are you able to stand for short periods of time?

PFA38 – Are you able to dry your back with a towel?
PFA39r1 – Are you able to run at a fast pace for two miles (3 km)?
PFA40 – Are you able to turn a key in a lock?
PFA41 – Are you able to squat and get up?
PFA42 – Are you able to carry a laundry basket up a flight of stairs?
PFA43r1 – Are you able to write with a pen or pencil?
PFA44 – Are you able to put on a shirt or blouse?
PFA45 – Are you able to get out of bed into a chair?
PFA47 – Are you able to pull on trousers?
PFA48 – Are you able to peel fruit?
PFA49 – Are you able to bend or twist your back?
PFA50 – Are you able to brush your teeth?
PFA51 – Are you able to sit on the edge of a bed?
PFA52 – Are you able to tie your shoelaces?
PFA53 – Are you able to run errands and shop?
PFA54 – Are you able to button your shirt?
PFA55 – Are you able to wash and dry your body?
PFA56 – Are you able to get in and out of a car?
PFB8r1 – Are you able to carry two bags filled with groceries 100 yards (100 m)?
PFB9 – Are you able to jump up and down?
PFB10 – Are you able to climb up five steps?
PFB11 – Are you able to wash dishes, pots, and utensils by hand while standing at a sink?
PFB12 – Are you able to make a bed, including spreading and tucking in bed sheets?
PFB13 – Are you able to carry a shopping bag or briefcase?
PFB14 – Are you able to take a tub bath?
PFB15r1 – Are you able to change the bulb in a table lamp?
PFB16r1 – Are you able to press with your index finger (for example, ringing a doorbell)?
PFB17 – Are you able to put on and take off your socks?
PFB18 – Are you able to shave your face or apply makeup?
PFB19r1 – Are you able to squeeze a new tube of toothpaste?
PFB20r1 – Are you able to cut a piece of paper with scissors?
PFB21r1 – Are you able to pick up coins from a tabletop?
PFB22 – Are you able to hold a plate full of food?
PFB23r1 – Are you able to pour liquid from a bottle into a glass?
PFB24 – Are you able to run a short distance, such as to catch a bus?
PFB25 – Are you able to push open a door after turning the knob?
PFB26 – Are you able to shampoo your hair?
PFB27 – Are you able to tie a knot or a bow?
PFB28r1 – Are you able to lift 10 pounds (5 kg) above your shoulder?
PFB29r1 – Are you able to lift a full cup or glass to your mouth?

PFB30 – Are you able to open a new milk carton?
PFB31r1 – Are you able to open car doors?
PFB32 – Are you able to stand unsupported for 10 minutes?
PFB33 – Are you able to remove something from your back pocket?
PFB34 – Are you able to change a light bulb overhead?
PFB36 – Are you able to put on a pullover sweater?
PFB37r1 – Are you able to turn faucets on and off?
PFB39r1 – Are you able to reach and get down a 5-pound (2 kg) object from above your head?
PFB40 – Are you able to stand up on tiptoes?
PFB41 – Are you able to trim your fingernails?
PFB42 – Are you able to stand unsupported for 30 minutes?
PFB56r1 – Are you able to lift one pound (0.5 kg) to shoulder level without bending your elbow?
PFC6r1 – Are you able to walk a block (about 100 m) on flat ground?
PFC7r1 – Are you able to run five miles (8 km)?
PFC13r1 – Are you able to run 100 yards (100 m)?
PFC21 – Are you able to run on even ground?
PFC29 – Are you able to walk up and down two steps?
PFC30 – Are you able to carry a suitcase up a flight of stairs?
PFC31 – Are you able to reach into a low cupboard?
PFC32 – Are you able to climb up 5 flights of stairs?
PFC33r1 – Are you able to run ten miles (16 km)?
PFC38 – Are you able to walk at a normal speed?
PFC39 – Are you able to stand without losing your balance for several minutes?
PFC40 – Are you able to kneel on the floor?
PFC41 – Are you able to sit down in and stand up from a low, soft couch?
PFC42 – Are you able to open a tight or new jar?
PFC43 – Are you able to use your hands, such as for turning faucets, using kitchen gadgets, or sewing?
PFC45r1 – Are you able to sit on and get up from the toilet?
PFC46 – Are you able to transfer from a bed to a chair and back?
PFC47 – Are you able to be out of bed most of the day?
PFC48 – Are you able to carry household items, such as heavy boxes or furniture, up a flight of stairs?
PFC49 – Are you able to water a house plant?
PFC51 – Are you able to wipe yourself after using the toilet?
PFC52 – Are you able to turn from side to side in bed?
PFC53 – Are you able to get in and out of bed?
PFM1 – Are you able to dig a 2-foot (1/2 m) deep hole in the dirt with a shovel?
PFM2 – Are you able to lift a heavy painting or picture to hang on your wall above eye-level?

PFM3 – Are you able to paint the walls of a room with a brush or roller for 2 hours without stopping to rest?
PFM4 – Are you able to row a boat for 30 minutes without stopping to rest?
PFM6 – Are you able to hand wash and wax a car for 2 hours without stopping to rest?
PFM7 – Are you able to complete 5 push-ups without stopping?
PFM9 – Are you able to rake leaves or sweep for an hour without stopping to rest?
PFM10 – Are you able to do a pull-up?
PFM12 – Are you able to lift a heavy object (20 lbs/10 kg) above your head?
PFM15 – Are you able to hit the backboard with a basketball from the free-throw line (13 ft/4 m)?
PFM16 – Are you able to pass a 20-pound (10 kg) turkey or ham to other people at the table?
PFM17 – Are you able to remove a heavy suitcase (50 lbs/25 kg) from an overhead bin on an airplane or bus?
PFM18 – Are you able to continuously swing a baseball bat or tennis racket back and forth for 5 minutes?
PFM19 – Are you able to complete 10 sit-ups without stopping?
PFM21 – Are you able to climb the stairs of a 10-story building without stopping?
PFM23 – Are you able to walk briskly for 20 minutes without stopping to rest?
PFM25 – Are you able to come to a complete stop while running?
PFM26 – Are you able to make sharp turns while running fast?
PFM27 – Are you able to jump rope for 10 minutes without stopping?
PFM28 – Are you able to jump over an object that is 1 foot (30 cm) tall?
PFM29 – Are you able to jump over a puddle that is 3 feet (1 m) wide?
PFM32 – Are you able to jump 2 feet (60 cm) high?
PFM33 – Are you able to walk across a balance beam?
PFM34 – Are you able to stand on one foot with your eyes closed for 30 seconds?
PFM35 – Are you able to walk in a straight line putting one foot in front of the other (heel to toe) for 5 yards (5 m)?
PFM36 – Are you able to put your hands flat on the floor with both feet flat on the ground?
PFM37 – Are you able to carry a large baby (15 lbs/7 kg) out of the house to a car or taxi?
PFM38 – Are you able to lift and load one 50-pound (25 kg) bag of sand into a car?
PFM40 – Are you able to climb a 6-foot (2 m) ladder?
PFM43 – Are you able to push an empty refrigerator forward 1 yard (1 m)?
PFM44 – Are you able to carry a 50 lb (25 kg) bag of sand 25 yards (25 m)?
PFM46 – Are you able to pull a sled or a wagon with two children (total 100 lbs/50 kg) for 100 yards (100 m)?
PFM49 – Are you able to stand up from a push-up position five times quickly?
PFM51 – Are you able to swim laps for 30 minutes at a moderate pace?
PFM53 – Are you able to dance energetically for an hour?

PFA1 – Does your health now limit you in doing vigorous activities, such as running, lifting heavy objects, participating in strenuous sports?
PFA2 – Does your health now limit you in exercising regularly?
PFA3 – Does your health now limit you in bending, kneeling, or stooping?
PFA4 – Does your health now limit you in doing heavy work around the house like scrubbing floors, or lifting or moving heavy furniture?
PFA5 – Does your health now limit you in lifting or carrying groceries?
PFA6 – Does your health now limit you in bathing or dressing yourself?
PFB1 – Does your health now limit you in doing moderate work around the house like vacuuming, sweeping floors or carrying in groceries?
PFB3 – Does your health now limit you in putting a trash bag outside?
PFB4 – Does your health now limit you in dancing for half an hour?
PFB5r1 – Does your health now limit you in hiking a couple of miles (3 km) on uneven surfaces, including hills?
PFB7 – Does your health now limit you in doing strenuous activities such as backpacking, skiing, playing tennis, bicycling or jogging?
PFB43 – Does your health now limit you in taking care of your personal needs (dress, comb hair, toilet, eat, bathe)?
PFB44 – Does your health now limit you in doing moderate activities, such as moving a table, pushing a vacuum cleaner, bowling, or playing golf?
PFB45 – Does your health now limit you in taking part in any sports (swimming, bowling, and so forth)?
PFB48 – Does your health now limit you in taking a shower?
PFB49 – Does your health now limit you in going for a short walk (less than 15 minutes)?
PFB51 – Does your health now limit you in participating in active sports such as swimming, tennis, or basketball?
PFB54 – Does your health now limit you in going OUTSIDE the home, for example to shop or visit a doctor's office?
PFC8 – Does your health now limit you in opening a previously opened jar?
PFC10 – Does your health now limit you in climbing several flights of stairs?
PFC11 – Does your health now limit you in doing yard work like raking leaves, weeding, or pushing a lawn mower?
PFC12 – Does your health now limit you in doing two hours of physical labor?
PFC35 – Does your health now limit you in doing eight hours of physical labor?
PFC36r1 – Does your health now limit you in walking more than a mile (1.6 km)?
PFC37 – Does your health now limit you in climbing one flight of stairs?
PFC54 – Does your health now limit you in getting in and out of the bathtub?
PFC56 – Does your health now limit you in walking about the house?
PFB50 – How much difficulty do you have doing your daily physical activities, because of your health?

Global06 – To what extent are you able to carry out your everyday physical activities such as walking, climbing stairs, carrying groceries, or moving a chair?

UAB MEDICINE | Orthopaedic Surgery

Brief Resilience Scale (BRS)

Please respond to each item by marking **one box per row**		Strongly Disagree	Disagree	Neutral	Agree	Strongly Agree
BRS 1	I tend to bounce back quickly after hard times	▢ 1	▢ 2	▢ 3	▢ 4	▢ 5
BRS 2	I have a hard time making it through stressful events.	▢ 5	▢ 4	▢ 3	▢ 2	▢ 1
BRS 3	It does not take me long to recover from a stressful event.	▢ 1	▢ 2	▢ 3	▢ 4	▢ 5
BRS 4	It is hard for me to snap back when something bad happens.	▢ 5	▢ 4	▢ 3	▢ 2	▢ 1
BRS 5	I usually come through difficult times with little trouble.	▢ 1	▢ 2	▢ 3	▢ 4	▢ 5
BRS 6	I tend to take a long time to get over setbacks in my life.	▢ 5	▢ 4	▢ 3	▢ 2	▢ 1

Scoring: Add the responses varying from 1-5 for all six items giving a range from 6-30. Divide the total sum by the total number of questions answered.

My score: ______ item average / 6

Smith, B. W., Dalen, J., Wiggins, K., Tooley, E., Christopher, P., & Bernard, J. (2008). The brief resilience scale: assessing the ability to bounce back. *International journal of behavioral medicine*, *15*(3), 194-200.

UAB MEDICINE | Orthopaedic Surgery

Percent of Normal Survey

How would you rate your affected joint/region today as a percentage of normal (0% to 100% scale with 100% being normal)?

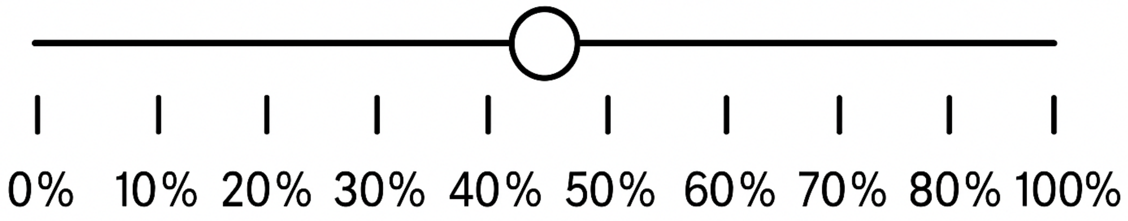

Supplement: Supplementary file 1 — Supplementary file1 (PDF 1266 kb) [file 590_2025_4558_MOESM1_ESM.pdf]
